# Aggregative Perivascular Tumor Cell Growth Pattern of Primary Central Nervous System Lymphomas Is Associated with Hypoxia-Related Endoplasmic Reticulum Stress

**DOI:** 10.7150/jca.54952

**Published:** 2021-05-05

**Authors:** Miaoxia He, Weiwei Zhang, Jianjun Wang, Lei Gao, Lijuan Jiao, Laixing Wang, Jianmin Zheng, Zailong Cai, Jianmin Yang

**Affiliations:** 1 Department of pathology, Changhai Hospital, Shanghai 200433, China.; 2 Department of Experimental Diagnose, Changhai Hospital, Shanghai 200433, China.; 3 Department of Hematology, Changhai Hospital, Shanghai 200433, China.; 4 Department of Neurosurgery, Changhai Hospital, Shanghai 200433, China.; 5 Department of Biochemistry and Molecular Biology, Second Military Medical University, Shanghai, 200433 China.

**Keywords:** Primary central nervous system lymphomas, APVT formation, clinical outcome, hypoxia, endoplasmic reticulum stress and signal transduction.

## Abstract

Primary central nervous system lymphomas (PCNSLs) often present a unique histopathological feature of aggregative perivascular tumor cells (APVT). Our previous studies showed that patients of PCNSL with APVTs exhibited poor long-term outcomes and increased expression of the endoplasmic reticulum stress (ERS) factor X-box-binding protein (XBP1). However, very little is known about molecular mechanism of the APVT formation in PCNSLs. The aim of this study is to determine if hypoxia-induced ERS is related to the APVT formation in PCNSLs. In this study, cell culture was used to observe the interplay between diffuse large B cell lymphoma (DLBCL) tumor cells and human brain microvascular endothelial cells (HBMECs) in different oxygen conditions. The expression of XBP1, CXCR and CD44 was manipulated by molecular cloning and siRNA technology. Mouse *in vivo* experiments and clinical studies were conducted to confirm our hypothesis. Our results showed that activated B-cell type-DLBCL cells easily migrated and invaded, and expressed high levels of XBP1 and stromal molecules CXCR4 and CD44 during hypoxia-induced ERS and dithiothreitol unfolded protein response (UPR). The gene upregulation (using overexpression vector) and downregulation (siRNA gene knock-out) in cultured cells and in mouse models further confirmed a close relation of the expression of XBP1, CXCR4, and CD44 with APVT formation, which is coincided with our clinical observation that increased expression of XBP1, CXCR4, and CD44 in the APVT cells in PCNSLs were associated with poor clinical outcomes. The results suggest that hypoxia-induced ERS and UPR might be associated with APVTs formation in PCNSL and its poor clinical outcomes. The results will help us better understand the progression of PCNSL with APVTs feature in daily pathological work and could be valuable for future target treatment of PCNSLs.

## Introduction

Primary central nervous system lymphoma (PCNSL) is an aggressive high-grade non-Hodgkin lymphoma of the central nervous system. There are two types of PCNSL: ABC (activated B-cell-like) and GCB (germinal center B-cell-like) 1-5. Most PCNSLs are ABC DLBCL (diffuse large B cell lymphoma) with poor prognosis and constantly present special clinical and pathological features 6-8. Histologically, malignant B-cells commonly accumulate around vessels, showing as aggregative perivascular tumor cells (APVT) 4, 9. This pattern is a characteristic pathology of PCNSL, but its pathogenesis is scarcely known 4, 8, 10-12. Our previous data revealed that the presence of APVTs was associated with increased tumor progression and shortened overall survival rate of the PCNSL patients, compared to those without APVTs 9. The expression of X-box-binding protein (XBP1) in APVT positive PCNSL cases was closely associated with poor prognosis of the patients 9. Our result is consistent with previous finding by Dr. Rubenstein group that XBP1 was upregulated in PCNSL, especially in tumor cells around tumor vessels 13. XBP1 is a major endoplasmic reticulum (ER) stress-associated transcriptional factor. The ER is responsible for protein biosynthesis, posttranslational modification and maturation. Meanwhile, unfolded proteins easily accumulate in the ER when cells are subjected to ER stress (ERS) induced by hypoxia, glucose deprivation, or certain chemicals 14, 15. These ER stressors activate the unfolded protein response (UPR), which leads to increasing release of unfolded or misfolded proteins participated in different aspects of tumor development and progression 15, 16. The most important transcriptional changes are mediated by inositol-requiring enzyme-1a (IRE1a) 17, 18. XBP1 is one of very important transcriptional factors in the IRE1a signal pathway and an important component of UPR 19. Upon ERS, IRE1a detects stress signal(s) caused by misfolded and/unfolded proteins that accumulate within the ER lumen, and mediates XBP1 mRNA maturation, leading to convert un-splicing form (XBP1u) to splicing form (XBP1s) that translocates into the nucleus and triggers the expression of ER stress response genes 17, 18. XBP1s shares the same protein structure with XBP1u except that it has extra 115 amino acids at its C-terminus due to reading frame shift of the gene after splicing 17. IRE1a and XBP1 can drive B-cell differentiation toward plasma cells and have been shown to contribute to multiple myeloma development 20. Many studies have shown that XBP1s is an essential survival factor under hypoxic condition and positively regulates tumor metastasis 16, 19, 21-23. Hypoxia can induce the expression of XBP1 at both transcriptional and translational levels 22. Brain tumor's environment is subject to several cellular stressors, including hypoxia 9, 11. Furthermore, we noticed that PCNSLs with an APVT growth pattern were more prone to disseminate intracranially than other brain tumors 9. We believe that hypoxia-induced ERS and UPR in PCNSL could increase the expression of XBP1 and other critical factors, which might have an important role in forming an APVT growth pattern in PCNSL 13. CXCL12 is a stromal cell-derived alpha chemokine member of the intercrine family with C-X-C motif. It can enhance tumor cell migration and invasion. It functions through C-X-C chemokine receptor type 4 (CXCR4). CD44 a cell-surface glycoprotein involved in cell-cell interactions, cell adhesion and migration. CXCL12/CXCR4 and CD44 could be involved in APVT formation and progression of PCNSLs. Therefore, it could be very interesting to see the role of these molecules in the cell-cell interactions, cell adhesion and migration of PCNSL cells.

In this study, we demonstrated that hypoxia-related ERS/UPR pathway activation was closely linked to APVT formation in PCNSLs. These findings may help us further understand the clinical pathological features and have potential usage in target treatment of PCNSLs in future.

## Materials and Methods

### Cell culture and cell interplay assays

GCB-DLBCL cell lines Ly1 and Ly8 courteously provided by Dr. B. Hilda Ye, Albert Einstein College of Medicine, NY were cultured in DMEM medium. Ly10 and HBL1 (ABC-DLBCL cell lines from ATCC, Manassas, VA, USA) was maintained in PRMI 1640 (GIBCO) medium. Both culture media were supplemented with 10% fetal bovine serum. The selection of DLBCL cell lines was purely dependent on their availability at the time of this study. All cells were maintained in 5% CO2 and 95% air in a normoxic humidified incubator at 37 °C. Human brain microvascular endothelial cells (HBMECs) (ScienCell Research Laboratories, Carlsbad, CA, USA) were cultured under the same condition using fibronectin-coated flasks. The cultures were replenished with fresh medium prior to each experiment. Hypoxic incubators with 3% O2 and 92% N2 were used to mimic hypoxic condition 14. The cell lines were mycoplasma free. The UPR was induced with 1 mM dithiothreitol (DTT) for 4 h as described in literature to create the UPR 24.

The cell interplay assays including cell adhesion, migration and invasion assays were performed according to methods previously reported literatures 25, 26. IRE1a, XBP1, CXR4 and CD44 mRNA expression levels were measured by qRT-PCR and proteins by Western blot. All *in vitro* experiments were performed at least three times.

### Construct gene silencing and expression vectors and establish stably gene-transfected cells

RNA silencing vectors, siXBP1 (siXBP1s/siXBP1), siCXCR4 and siCD44, were constructed in expressible lentiviral vector (PL/U6/shRNA/GFP) following procedures described in literatures 27. Three siRNA lentiviral vectors for each target gene were constructed. One with the highest inhibition rate was selected for *in vitro* and *in vivo* experiments. siRNA primers for the target genes were designed by using Thermo-Fisher Scientific online tool BLOCK-iT™ RNAi Designer. The oligonucleotide sets for constructing selected lentiviral silencing vectors used in this study were: CACCGCCTGTCTGTACTTCATTCAACGAATTGAATGAAGTACAGACAGGC (F, forward) and AAAAGCCTGTCTGTACTTCATTCAATTCGTTGAATGAAGTACAGACAGGC (R, reverse) for siXBP1, CACCGTCCTGTCCTGCTATTGCATTACGAATAATGCAATAGCAGGACAGGA (F) and AAAATCCTGTCCTGCTATTGCATTATTCGTAATGCAATAGCAGGACAGGAC (R) for siCXCR4, and CACCGCCGTTGGAAACATAACCATTACGAATAATGGTTATGTTTCCAACGG (F) and AAAACCGTTGGAAACATAACCATTATTCGTAATGGTTATGTTTCCAACGGC (R) for siCD44. The target sequence of XBP1 siRNA is located in a common area of XPB1 and XBP1s, which silences both forms of XBP1. The constructed siRNA lentiviral vectors and control lentiviral vector were respectively co-transfected with lentiviral packaging plasmids (Packaging Mix K4975 from Invitrogen) into HEK 293T cells by using Lipofectamine 2000 (Invitrogen) to produce infectable siRNA lentiviral particles. The infectable siRNA lentiviruses were harvested and then infected cell lines Ly1, HBL1 and HUMEC to create stable cell lines. These transduced cells were verified for the presence of correct vectors and their silencing capabilities. The silencing efficiencies of siXBP1, siCXCR4 and siCD44 vectors selected for this study were 79.37%, 68.43% and 46.29%, respectively.

We also constructed XBP1s high expression lentiviral vector with doxycycline inducible promotor by cloning into pTetOne-XBP1s-GFPa1-Puro vector and created stably transfected XBP1s expression cell lines according to the procedures described in previously published papers 28.

### Cell Adhesion, Migration and Invasion Assays

To determine the adhesion activity of DLBCL cells to HBMEC cells, the wash assay was used 26, 29. HBMEC cells were planted into Costar 24-well cell culture plates (Corning Life Sciences, Tewksbury, MA, USA) (about 80% confluence) and cultured overnight. DLBC lymphoma cell line cultures were replenished one day before the test. Next day, 5 x 10^5^ DLBC lymphoma cells were seeded into each well. The plates were spun down at 300 x g for 10 min at room temperature and then continued to be cultured for 1 h. At the end of culture, the culture medium was carefully removed and the plates were washed gently with PBS (phosphate-buffered saline) 3 times without disrupting adhered cells. The plates were observed under a microscope for cell adhesion.

For cell migration assay, Costar 24-well transwell plates with 8 µm pore size ((Corning Life Sciences, Tewksbury, MA, USA) were used according to manufacturer's instruction and previously published literatures 25, 30. A total of 800 µl medium containing 2% FBS and 10 ng/ml CXCL12 was added to each lower chamber of wells. Each upper chamber (insert well) were filled with 200 µl of DLBCL cell suspension at 10^5^ cells/ml. The plates were cultured for 24 h. Cells migrated to lower chambers were observed under a microscope.

In the cell invasion assay on Costar 8 µm pore size 24-well transwell, Corning® Matrigel® Growth Factor Reduced (GFR) Basement Membrane Matrix (Cornig Life Sciences, Tewksbury, MA, USA) was coated on the membrane of inserts (upper chambers) according to manufacturer's instruction. Tumor cells were cultured in 10% FBS DMEM or PRMI 1640 until reached the density of 1×10^5^/ml, then fed with serum-free medium and seeded into the coated upper chamber of the transwell plates. The bottom chambers of the plates were added with DMEM containing 10% FBS and CXCL12 at the concentration of 100 ng/ml. The cells were cultured for 48 hours to allow cells in the upper chamber to travel through extracellular matrix-coated porous membrane. Images of the lower chambers were captured on an Olympus inverted microscope under a 10× objective 25, 30, 31.

### PCNSL Clinical Case Study

A total of 56 patients with PCNSL retrospectively enrolled in this study were diagnosed and treated in Changhai Hospital over a 10-year period (2004 to 2014). No patients had prior hormone treatment before biopsy. Clinical pathological characteristics of the patients were listed in Table 1. The control group included 10 cases of primary nodal DLBCL and 10 cases of glioblastoma. Patients with serological HIV positive or those who received immunosuppressing drugs before surgery were excluded. The patients were treated with high-dose methotrexate chemotherapy (dose, ≥1 g/m^2^) or with a median dose radiotherapy at of 34+12 Gy applied to the whole brain. The percentages of the patients with different therapies in APVT positive and APVT negative groups were similar (no statistical significance). The clinical prognostic variables were defined by the International Extranodal Lymphoma Study Group (IELSG) for PCNSL 32. All patients had written consent for sample collection and research use. The study was performed in accordance with the Declaration of Helsinki and was approved by the local ethics review committee.

Follow-up information was obtained from the hospital information system and the patients or patients' relatives. Overall response rate (ORR) was defined as the percentage of patients whose lymphoma remitted after the routine anti-tumor treatment. Overall survival (OS) was calculated from the date of pathological diagnosis to death or to the last date of follow-up 33.

All specimens were formalin fixed and paraffin embedded (FFPE). Slides for the diagnosis were reviewed by three pathologists in accordance with WHO classification 4, 7, 8. APVT evaluation and IHC staining were evaluated as described in our previous publication 9. Primary antibodies CD20, CD3, IRE1a, XBP1, CXCR4, and CD44 used were purchased from Dako (Hamburg, Germany).

### Animal experiment

Animal experiments were approved by the Institutional Animal Care and Use Committee of the University. BALB/c nude mice were purchased from SLAC Laboratory Animal Company, Shanghai, China. Five-to seven-weeks old male nude mice were randomly divided into 5 groups. Five different types of tumor cells (original ABC-DLBCL HBL1 cell line, mock transected cells with the control lentiviral vector alone and 3 stably transferred with siRNAs: HBL1-XBP1s/*si*XBP1, HBL1-*si*CXCR4, and HBL1-*si*CD44, each with 2×10^6^ cells) were injected into the brains of above 5 corresponding groups of mice through the head midline at an intended depth of 0.5 cm under anesthesia intraperitoneally with 5% chloral hydrate. At least 10 mice were used per experimental group. As control, cells were injected subcutaneously and observed for the tumor cell growth pattern. The same experimental procedures were used to generate the GCB-DLBCL brain xenograft mouse model using untreated Ly1 cells, mock transfected Ly1 and 3 different siRNA stably transferred Ly1 cells (Ly1-XBP1s/*si*XBP1, Ly1-*si*CXCR4 and Ly1-*si*CD44). Mice were daily monitored for neurological symptoms and signs (cognitive and motor impairments such as ataxia, hemiplegia, and weight loss) and were provided necessary care to reduce any suffering from experiments. The animals were euthanized 50 days after cell injection as the maximum growth period. Samples were collected for other tests 9, 34.

### Statistical Considerations

Prognostic factors were analyzed in accordance with the IELSG (International Extranodal Lymphoma Study Group) prognostic score. The clinical characteristics, pathological features and immunohistochemically scores (Allred score) of the patients in different subgroups were compared by using the *X*^2^ test for categorical variables 24. All of the probability values were two sided and were considered to be statistically significant when P<0.05. Survival curves were generated using the Kaplan-Meier method. Overall survival curves were compared using the log-rank test. Each patient was assigned to a group in accordance with the pathological features or immunohistochemical parameters. Analyses were performed using SPSS, version 23.0, statistical package for Windows (Stat Soft, Inc., Tulsa, USA).

## Results

### Hypoxia increased ABC-DLBCL cell migration, invasion and adhesion to HBMECs

**Figure 1:**
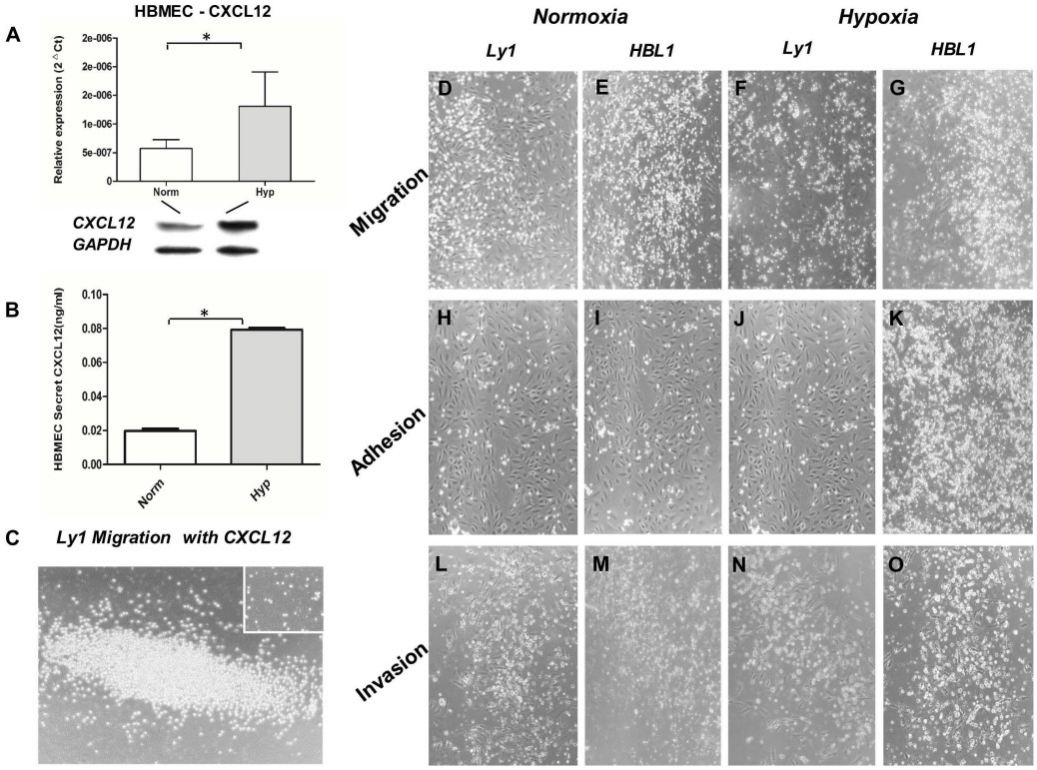
** Interplay between DLBCL tumor cells and HBMECs under normoxic (Norm) and hypoxic (Hypo) condition.** HBMECs increased the expression and secretion of CXCL12 under hypoxic culture condition detected by qRT-PCR (Panel A upper part), Western blot (Panel A lower part) and ELISA (Panel B). CXCL12 promoted tumor cell (ly1) migration under hypoxia (Panel C) compared to the negative control without CXCL12 in the culture (small insert in the figure). Panel D, H and L are the results Ly1 cell migration, adhesion and invasion under the normoxia condition, respectively. Panel E, I and M are the results HBL1 cell migration, adhesion and invasion under the normoxia condition, respectively. Panel F, J and N are the results Ly1 cell migration, adhesion and invasion under the hypoxia condition, respectively. Panel G, K and O are the results HBL1 cell migration, adhesion and invasion under the hypoxia condition, respectively. Increased migration, adhesion and invasion were more notable in the ABC-DLBCL cells (HBL1, Panel G, K and O) than in the GCB-DLBCL cells (Ly1, Panel F, J and N) under a hypoxic environment. There was no significant difference in the invasion of GCB tumor cells (Ly1) between normoxic (Panel D, H and L) and hypoxic culture condition (Panel F, J and N). The impact of hypoxia on cell proliferation, adhesion and invasion of lymphoma cells was semi-quantitated by vision estimating cell density on micro-images of cell cultures.

We measured CXCL12 expression in HBMECs under normoxic and hypoxic condition by qRT-PCR, Western Blot and ELISA assay. It was found that the expression (mRNA and protein) of CXCL12 was more than doubled under hypoxic condition (Figure 1A) while the expression of VEGF was just slightly increased (data not shown). The increased CXCL12 protein was further confirmed by ELISA (Figure 1B). When the Ly1 tumor cells were co-cultured with HBMECs under hypoxic condition, chemokine CXCL12 significantly promoted tumor cell migration (Figure 1C). We concluded that under hypoxia, HBMEC expressed more CXCL12 that promoted DLBCL tumor cells to migrate and adhere to vessels.

To test if the formation of APVTs in PCNSLs could be regulated by a brain special ER microenvironment, we observed the interaction between HBMECs and different GCB-DLBCL (Ly1 and Ly8) or ABC-DLBCL (Ly10 and HBL1) cell lines in normoxic and hypoxic condition. Neither migration nor invasion was observed for tumor cells when cultured with the serum under normoxia and hypoxia culture condition. When the tumor cells and HBMECs were cultured together, tumor cell migration in hypoxic condition (Figure 1F and G) was much obvious than in normoxic condition (Figure 1D and E). Meanwhile, more tumor cells and HBMECs adhered together under hypoxic (Figure 1J and K) than normoxic condition (Figure 1H and I). They had were more in hypoxic (Figure 1Nand O) than in normoxic condition (Figure 1L and M). In addition, ABC-DLBCL tumor cells (DHL1) had higher activities of the migration, and invasion and adhesion to HBMECs than GBC-DLBCL cells (Ly1) under hypoxic condition though both of cell types had increased activities.

### Hypoxia and UPR activate XBP1 pathway molecules in ABC-DLBCL

**Figure 2:**
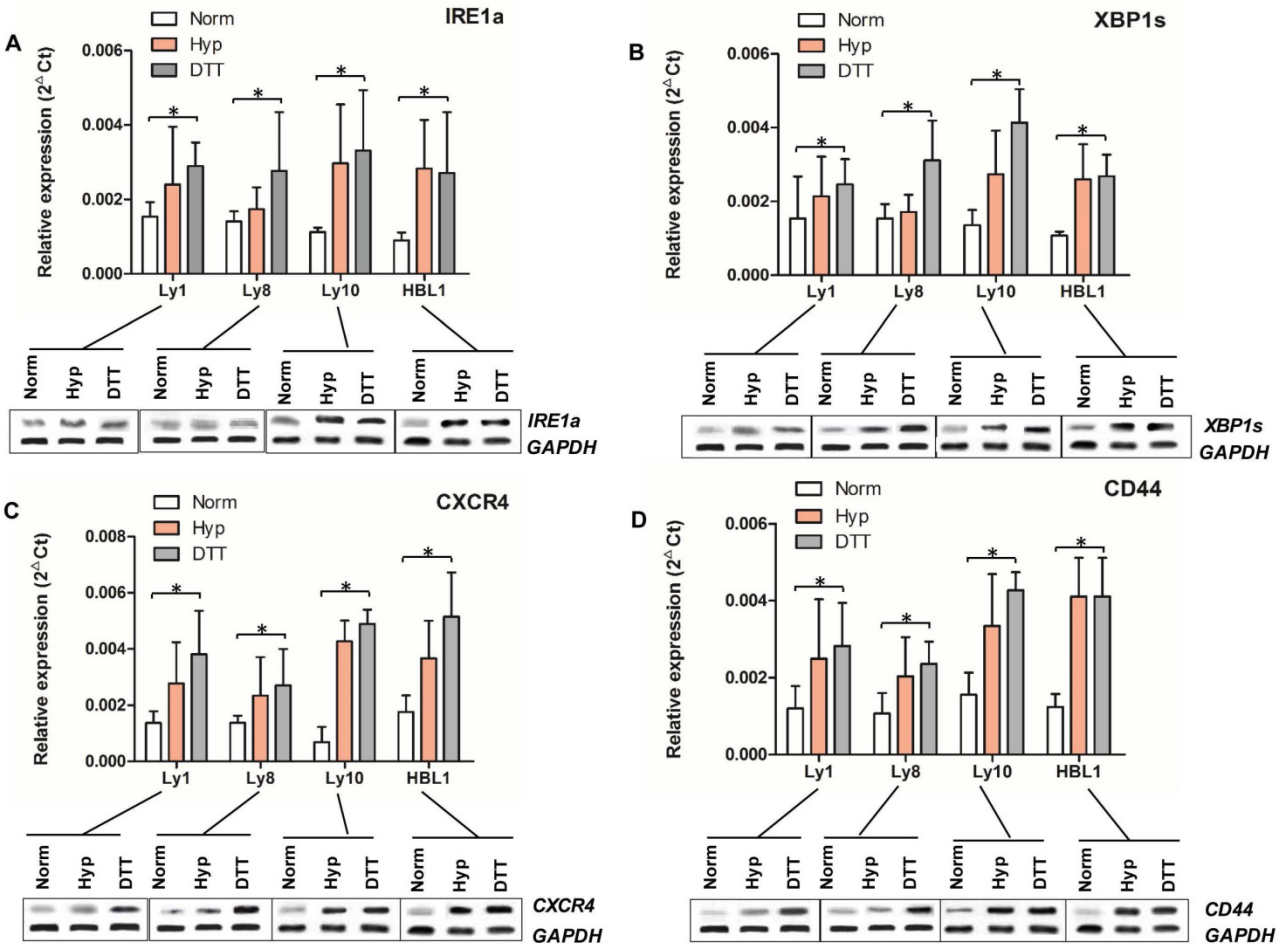
** mRNA and protein expression of IRE1a, XBP1, CXCR4, and CD44 in DLBCL cell lines.** mRNAs and proteins of IRE1a (Panel **A**), XBP1s (Panel **B**), CXCR4 (Panel **C**), and CD44 (Panel **D**) in DLBCL cell lines cultured under different conditions were determined by qRT-PCR (bar graphs) and Western blot (protein bands at the bottom of each panel). The expression of all these four molecules in ABC and GCB tumor cells was upregulated under hypoxia and DTT-induced UPR condition compared to those under normoxic condition (**P<0.5**).

IRE1a¸ XBP1s, CXCR4 and CD44 levels in different DLBCL cell lines under normoxic and hypoxic condition were measured by qRT-PCR and Western Blot. There was no significant difference in the average expression levels of all these 4 molecules between two types of DLBCL cell lines under normoxic condition except for the relatively low level of CXCR4 in Ly10 cells. Hypoxic condition upregulated the expression of IRE1a, XBP1s, CXCR4 and CD44 in all cell lines tested. The expression in ABC cell lines was higher than that in GCB cell lines under hypoxic condition and after DTT induction (Figures 2A-D).

**Figure 3:**
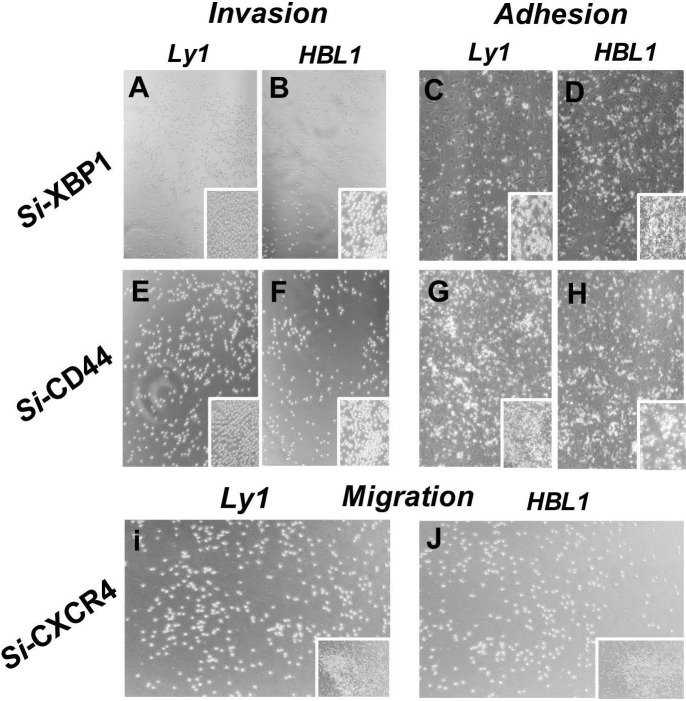
** Effects of silencing IRE1a, XBP1, CXCR4, and CD44 genes on the invasion, adhesion and migration of lymphoma cells.** IRE1a, XBP1, CXCR4, and CD44 genes in lymphoma cells were silenced by infecting the tumor cells with lentiviruses carrying siRNAs specific for these genes. The invasion, adhesion and migration of these cells together with their parental cells were assessed. XBP1 and CD44 silencing reduced the tumor cell invasion and adhesion to HBMECs (Panel A-H). CXCR4 silencing reduced the tumor cell migration (Panel I and J). Inserts in Panel J-O are vector alone cell controls. The impact of hypoxia on cell proliferation, adhesion and invasion of lymphoma cells was semi-quantitated by vision estimating cell density on micro-images of cell cultures.

To extend these findings, we tested whether knockout of XBP1, CXCR4, and CD44 genes by siRNA technology affected the interaction between DLBCL cells and HBMECs. Ly1 and HBL1 cells stably infected with lentiviral vectors carrying the small interfering RNA (siRNA) of XBP1, CXCR4, or CD44 or lentiviral control vector were tested. The impact of culture condition and gene silencing on cell proliferation, adhesion and invasion of lymphoma cells was semi-quantitated by vision estimating cell density on micro-images of cell cultures. It was found that silencing XBP1 (Figure 3A-D) and CD44 (Figure 3E-H) genes reduced the invasion of both cell lines compared to non-silenced control cells (insert in each panel). Meanwhile, silencing CXCR4 significantly reduced the migration of these tumor cells (Figure 3 I and J). These data indicate that XBP1, CXCR4, and CD44 play a significant role in the interplay between tumor cells and endothelial cell HBMECs under hypoxia-related ERS/UPR special microenvironments.

### Expression of XBP1s promotes APVT formation and tumor growth in DLBCL brain xenograft models

Given the increased expression of XBP1, CXCR4, and CD44 during ERS/UPR activation under hypoxic condition in DLBCLs, we further investigated possible contribution of this pathway to the tumor APVT growth pattern. Ly1 (GCB-DLBCL) and HBL1 (ABC-DLBCL) cells were stably transduced with doxycycline-inducible XBP1s gene expression vector 35. These cells were then transplanted into the flanks of the brain of immunodeficient BALB/c mice 9. Meanwhile, untreated Ly1 and HBL1 cells were transplanted into the same brain locations of control mice. Non-transplanted nude mice were also served as blank controls.

**Figure 4:**
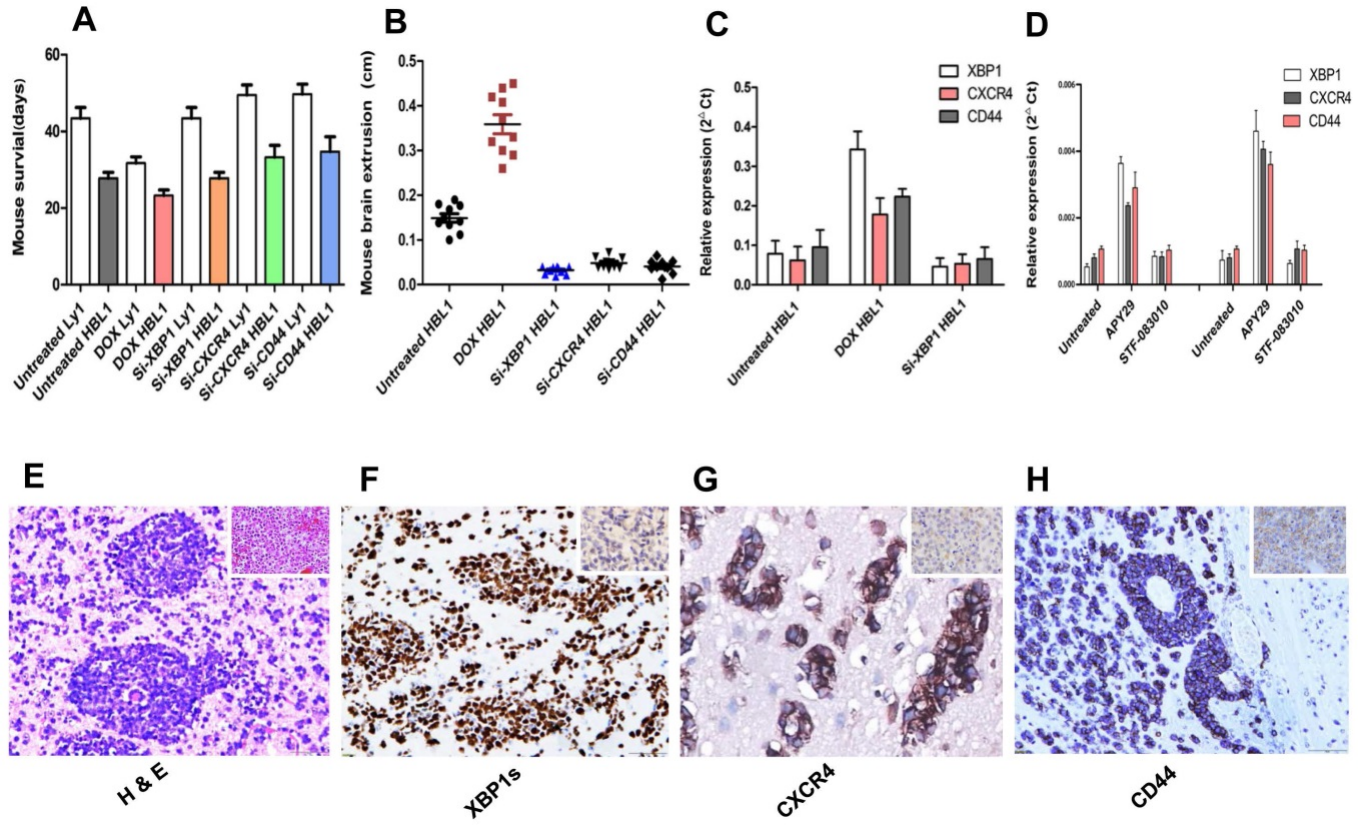
** Expression of XBP1s promotes APVT formation and tumor growth in mouse brain DLBCL xenograft models.** BALB/c nude mice brains were inoculated with DLBCL cell lines stably transfected with different vectors to create different brain DLBCL xenograft models. The impact of XBP1, CXCR4 and CD44 on the tumor morphology, the gene expression and the survival was investigated. Panel A: Overall survival of mice inoculated with DLBCL cells stably transfected with doxycycline inducible XBP1s expression vector or one of gene silencing vectors for XBP1, CXXR4 and CD44 with or without doxycycline treatment. Results shown that the mice inoculated XBP1, CXXR4 or CD44 gene silenced DLBCL xenograft tumors lived longer that those inoculated with unsilenced tumor cells under the same treatment with doxycycline (DOX). There is statistically significant difference in average survival time between the mice inoculated silenced and unsilenced tumor cells (P< 0.05). Panel B: The mouse brain extrusion degree. Panels C: qRT-PCR detection of the expression of XBP1, CXCR4 and CD44 in tumor tissues from xenograft mice with different treatments. Panel D: Expression of XBP1, CXCR4 and CD44 in cultured cells in the presence of XBP1 activator APY29 (100 µM) and suppressor STF-083010 (100 µM) by qRT-PCR. These concentration activated and inhibited about 85% of IRE1a activity, respectively. Panel E**:** A typical APVT growth pattern in the xenograft mouse brain biopsy samples (HE×400). Panel F-H**:** Immunohistochemical staining displayed that the APVTs in the mouse brain xenografts were positive for XBP1s, CXCR4, and CD44 in the doxycycline HBL1 group (IHC×400).

All untreated normal blank control nude mice survived to the maximal observing period of 50 days. After 21 days, tumor bearing mice started having neurological and physical condition, including increased intracranial pressure with eye bleeding and brain extrusion, weight loss and cachexia. Most mice suddenly died before we scheduled to euthanize them at the maximal observing period (Figures 4A-B). Administration of doxycycline promoted the growth of both ABC and GCB xenograft tumors carrying doxycycline-inducible XBP1s gene and reduced their grafted mouse overall survival (Figures 4A). As expected, the analysis of postmortem tumor biopsies showed APVT features formed more than 5 APVTs/ low power field (LPF) in median of both brain xenograft tumor models (Figures 4E). Doxycycline induction significantly increased the expression of XBP1s, CXCR4 and CD44 detected by qRT-PCR (Figure 4C) and by immunohistochemistry (Figure 4F-G). DLBCL xenograft tumors with the special APVT feature were easy to disseminate to and invade other tissues without obvious tumor mass formation at original sites, which really differed from that of the glioma brain model 34. Meanwhile, we transplanted *si*XBP1, *si*CXCR4, and *siCD44* lentivirus-transfected Ly1 and HBL1 cells into the brain xenograft model. The siRNAs negatively impacted the tumor growth (Figure 4B) and reduced APVT counts to less than 2 APVTs/LPF. These mice lived longer than those inoculated with control ABC and GCB tumor cells (Figures 4A). The expression of XBP1s, CXCR4, and CD44 in tumor biopsy samples was downregulated in siRNA lentivirus-transfected xenografts (Figure 4C).

XBP1 protein level was also monitored in the presence of APY29, a XBP1 activator and STF-083010, a XBP1 suppressor, which actions are through activating and inhibiting IRE1a, respectively 35. We found that 100 µM of APY29 significantly upregulated XBP1 expression in both ABC-DLCBL and GCB-DLBCL cells, which in turn promoted CXCR4 and CD44 expression, while the inhibition by the same concentration of STF-083010 was not obvious (Figure 4D).

### Increased expression of XBP1 pathway molecules in PCNSL with APVTs and its correlation with poor prognosis of patients

**Figure 5:**
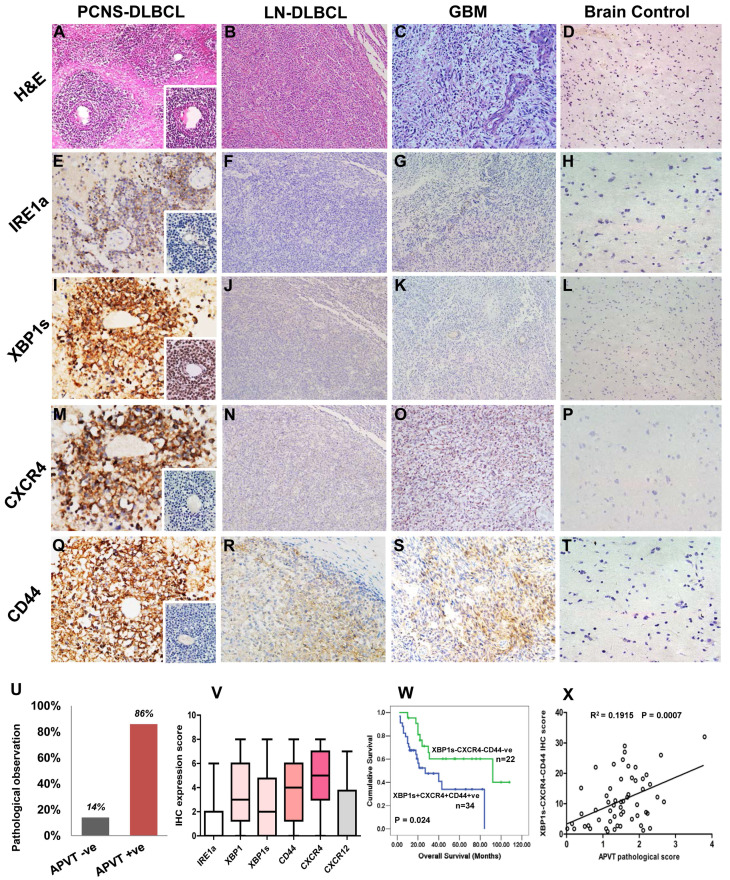
**Immunohistochemical detection of XBP1 pathway molecules in PCNSLs with APVTs and their correlations with clinical outcomes.** Biopsy tissue samples from patients were fixed with neutral formalin and embedded within paraffin blocks (FFPE). The tissue slides (4 μm thick) were cut from these FFPE blocks and used for H&E staining and immunohistochemistry study. Panel A shows H & E stained multiple layers of medium-to-large tumor cells surrounding vessels, forming an APVT growth pattern in PCNSLs, not in the nodal DLBCL, glioblastoma multiforme (GBM), and brain control tissue (Panels B-D). Panels E-T present positive immunostainings of IRE1a, XBP1 (XBP1s), CXCR4, and CD44 staining in the PCNSLs, compared with negative or weak staining in lymph node DLBCL, glioblastoma and brain tissue. The original magnification of all microscopic image was x200. Panel U indicates high rate (85%) of APVT presence in PCNS-DLBCL cases, Panel V reveals high immunostaining scores of the XBP1, XBP1s, CXCR4 and CD44 expressions and relatively low score for IRE1a and CXCL12 expressions. Panel W demonstrates shorter OS time of the cases with XBP1s-CXCR4-CD44-positive staining than that of the cases with negative staining. A correlation between APVT and XBP1-CXCR4-CD44-positive staining is shown in Panel X.

To extend our previous PCNSL study 9, we further analyzed the APVT special features and focused on the expression of additional molecules associated with XBP1 pathway in 56 newly recruited PCNSL patients. APVT pattern (Figure 5A) was found in the majority of PCNSL cases, but not in nodal DLBCL (Figure 5B), glioblastoma (GBM, Figure 5C) and normal brain tissue (Figure 5D) (Table 1) 9. Primary treatment with high-dose methotrexate or with radiation therapy, sex, ages and other serological markers, such as LDH, β_2_-microglobulin, did not affect ORR and three-year overall survival OS (Table 1).

Immunostaining showed that IRE1a expression was slightly increased in APVTs of PCNSLs (Figure 5E). However, the expression of XBP1s, CXCR4, and CD44 was much higher in APVT tumor cells of PCNSL than those in the control tissues, which were negative except for very weak positive for CD44 (Figure 5I-T). Overall survival rate for PCNSL patients with APVTs was statistically significantly shorter than those without APVTs as described in our previous study (Figure 5U) 9. The immunohistochemical scores are shown in Figure 5V. The OS of the cases with XBP1s, CXCR4, and CD44 positive staining was shorter than that of the negative cases (log-rank test, P=0.024) (Figure 5W). A positive correlation was noticed between the APVT pathological score and IHC score of XBP1-CXCR4-CD44 (R^2^=0.0775, P=0.0374, Figure 5X).

## Discussion

This study focused on possible involvement of the microenvironment in the PCNSL APVT formation using representative GCB- and ABC-DLBCL lymphoma cell lines, and HBMECs. Our results showed that the tumor cell migration was more obvious in hypoxia than in normoxic condition. The ABC-DLBCL tumor cells were more invasive than the GCB-DLBCLs under hypoxia. HBMECs promoted tumor cell migration and adhesion under hypoxic condition through producing increased CXCL12 expression. Since CXCL12 could enhance tumor cell migration and invasion, we believe that tumor cells' ability of the migration and invasion was partially due to increased expression of CXCL12 by HBMECs under hypoxia. Furthermore, we evaluated the expression of CXCL12 and its receptor CXCR4 as well as some XBP1 pathway molecules in tumor cells under different culture condition. It was found that except for the low level of IRE1a in DLBCL cells, the expression of XBP1s, CXCR4 and CD44 was significantly upregulated under a hypoxic culture condition. This result is consistent with other groups' investigation that XBP1s can be upregulated under hypoxia 36, 37. Our previous study showed that patients of PCNSL with APVTs exhibited poor long-term outcomes and had increased expression of ERS factor XBP1 9. The results were further confirmed in this study by using newly recruited PCNSL cases. Therefore, we concluded that under hypoxic condition the DLBCL tumor cells experienced ERS, which led to increased tumor cell migration and adherence to blood vessels. This event was closely associated with XBP1 and the micro-environmental stromal molecules CXCR4 and CD44. Comparing the GCB-DLBCL cells (Ly1 and Ly8), the invasion and migration activities of ABC-DLBCL cells (Ly10 and HBL1) were more notable. ERS usually leads to UPR and XBP1 splicing. When UPR was induced using DTT under normoxia, mRNA and protein levels of XBP1s, CXCR4, and CD44 were increased in DLBCL cells, which is similar to the results found under hypoxia (Figure 2A). Knocking down XBP1, CXCR4, and CD44 by siRNA technology altered the interplay between tumor cells and HBMECs. Silencing XBP1 and CD44 reduced tumor cell invasion and adhesion to HBMECs, whereas silencing CXCR4 decreased the tumor cells migration. In the brain DLBCL xenograft mouse model, in which the expression of XBP1, CXCR4, and CD44 were manipulated by doxycycline and/or siRNA-silencing, we observed a close association between APVT formation and these molecules' expression in different xenografts. Since XBP1 can bind to promoter regions of CXCR4 and CD44 in our chromatin immunoprecipitation (ChIP) assay and increase the expression of those two molecules (data not shown), we conclude that XBP1(s) has played a key role in PCNSL APVT formation and tumorigenicity. Different subtypes of DLBCL cell lines, even different cell lines in the same subtype (GCB-DLBCLs) could be different in the expression of XBP1s and IRE1 as Bujisic and his colleagues presented in the published paper 38. In addition, DLBCLs in the central nerve system are different from peripheral DLBCLs in their growth and metastasis 4, 8. XBP1, CXCR4, and CD44 were expressed in perivascular areas with a broad distribution within PCNSL. The prognostic values of APVTs were more evident in the patients when it was combined with the immunostaining results of XBP1, CXCR4, and CD44. The OS of the cases positive for XBP1, CXCR4, and CD44 was shorter than that of the negative cases. These results indicate that the high-level expression of XBP1, CXCR4 and CD44 is closely correlated with APVTs, implying a poor prognosis of the patients.

Clinical studies have shown that the low oxygen tension in a neoplastic lesion is an independent indicator of poor outcome and is correlated with increased metastatic risk 14, 21, 39. It is well known that there is a relative higher oxygen level in brain than in most of organs of the human body due to its high oxygen demand. Therefore, relatively hypoxia could happen at certain circumstance especially in a rapid growing tumor. In fact, hypoxia has been proposed to an important cellular stressor in the brain tumor environment 14, 39, 40. In addition, the microenvironment of tissue/cells close to blood vessels will have relatively higher oxygen level than the areas distant to blood vessels. As a result, the distant mobile cells (such as lymphocytes) could migrate to relative high oxygen areas close to the vessels in a gradient oxygen environment and/ the presence of stimulating factor(s). This could be more pertinent when a fast expanded tumor leads to hypoxic condition in tumor tissue 40. Severe hypoxia activates ERS and UPR to induce vascular cells to produce additional crucial factors that attract tumor cells and promote their migration, adhesion and invasion 14. These migrated tumor cells become more aggressive than surrounding tumor cells form APVTs. This is a unique characteristic of PCNSL cells and is absent in any other brain tumor, such as the glioma.

Although the IRE1a/XBP1 pathway represents the most critical ERS response and UPR pathway 13, 23, 34, our results showed XBP1 alone could upregulate the expression of CXCR4 and CD44. The association between XBP1 status and the CXCR4 and CD44 expression suggests that IRE1a activation may not be the main response in this hypoxia model. Or the current relatively low level of IRE1a was enough to promote XBP1 mRNA splicing. XBP1 overexpression could be induced by other factors rather than IRE1a alone. The high level of XBP1 can promote its own activation under the hypoxia-associated ERS and UPR.^22^ Thus, the enhanced expression of XBP1 and stromal molecules CXCR4 and CD44 can be sufficient for initiating the pathway, triggering tumor APVT growth, and increasing tumor aggressiveness. Although we did not observe the inhibition of tumor cell migration by an anti-CXCL12 antibody we tested (data not shown) we could not rule out the role of CXCL12 in hypoxic condition. The antibody we used was not a confirmed neutralizing antibody and might not be able to block CXCL12's function. We further found that the interference of the expression of CXCL12 receptor CXCR4 in tumor cells inhibited DLBCL tumor cell migration. Consistent with this notion, HBMECs appeared to express additional CXCL12 in a hypoxic environment. Such an upregulation can promote the migration and invasion of DLBCL tumor cells. Therefore, inhibiting CXCR4 expression in DLBCL tumor cells can prevent their migration and invasion. Meanwhile, the CXCR4 and CD44 expression was significantly upregulated in DLBCL tumor cells under hypoxic condition. CD44 is an adhesion molecule present on the surface of lymphocytes and promotes lymphocyte homing and binding to the endothelial vein 41-43. The high expression of CD44 in hypoxic tumor regions is plausible because hypoxia promotes CXCR4-mediated cell homing 42-44. An augmented CXCR4 signal was translated into increased vessel sprouting and angiogenesis in various assays 43, 44. The CXCR4 expression on tumor cells is upregulated by hypoxia and by other angiogenic factors, which are capable of directing the trafficking of normal and malignant cells expressed high levels of CXCL12 and CD44 43-45. The clinical relevance of hypoxia-associated ERS and UPR has been studied in a variety of tumors. Our clinical data further demonstrated that the hypoxia-associated ERS and UPR increased the expression of XBP1, CXCR4 and CD44. Increased expression of these molecules was associated with APVT formation in mice and with poor prognosis in patients with PCNSL. These findings provide novel insights into how microenvironment influences multi-component complexes to regulate key aspects of tumor growth and progression 41, 43. The results from this study could have potential application. Since XBP1 is a key molecule affecting tumor APVT formation, tumor growth and clinical prognosis of PCNSL patients, small molecules targeting this molecule might be able to prevent growth of the malignancy and improve clinical outcomes. In addition, our results indicate that hypoxia could cause to the cascade of reactions, leading to the increased expression of XBP1 and accelerated tumor growth. Further investigation of possible impact of hyperoxia on the growth of PCNSLs and clinical outcome could be very interesting and valuable.

## Figures and Tables

**Table 1:** Clinical pathological characteristics of 56 patients with PCNSL.

Characteristics	PCNSL (%)	P *value*
No.	56	
**Ages (Years)**		
Range	25-71	
<60	22 (39)	<0.05
>60	34 (61)	
**Gender**		
Male	31 (55)	>0.05
Female	25 (45)	
Multiple lesions	12/56 (21)	
Deep structural involvement	6/56 (11)	
Ocular lesion	3/56 (5)	
CSF cytology examination	7/56 (13)	
**LDH (lactate dehydrogenase) level**		
Normal	30 (54)	>0.05
High	26 (46)	
**β2 microglobulin**		
Normal	31 (55)	>0.05
High	25 (45)	
**APVT**		
Positive	48 (86)	<0.01
Negative	8 (14)	
IELSG score*		
Low (0-1)	10 (18)	<0.05
Intermediate (2-3)	22 (39)	
High (4-5)	24 (43)	
High-dose methotrexate	26/56 (46)	
ORR (overall response rate)	16/56 (29)	
3- years OS (overall survival)	20/56 (36)	

* IELSG: International Extranodal Lymphoma Study Group. Deep structural involvement, such as that in the corpus callosum and/or basal ganglia and/or brainstem and/or cerebellum.

## Abbreviations

PCNSL: primary central nervous system lymphoma
APVT: aggregative perivascular tumor cells
ER: endoplasmic reticulum
ERS: endoplasmic reticulum stress
XBP1: X-box-binding protein 1
XBP1u: XBP1 un-splicing form
XBP1s: XBP1 splicing form
IRE1a: inositol-requiring enzyme-1a
HBMECs: human brain microvascular endothelial cells
DLBCL: diffuse large B-cell lymphoma
C-X-C: chemokine receptor
UPR: unfolded protein response
siRNA: small interfering RNA
ABC: activated B-cell-like
GCB: germinal center B-cell-like
DTT: dithiothreitol
PCR: polymerase chain reaction
qRT-PCR: quantitative reverse transcription PCR
HIV: human immunodeficiency virus
Gy: Gray, an international unit of radiation
IELSG: international extranodal lymphoma study group
ORR: overall response rate
OS: overall survival
FFPE: formalin fixed and paraffin embedded
WHO: world health organization
IHC: immunohistochemical
LDH: lactate dehydrogenase
ChIP: chromatin immunoprecipitation
